# Catatonia associated with mosaic 18q deletion syndrome: a case report

**DOI:** 10.1186/s12888-026-08292-7

**Published:** 2026-06-18

**Authors:** Angela Luzia Herscheid, Christiana Franke, Nicoleta Carmen Cosma

**Affiliations:** 1 Department of Psychiatry and Psychotherapy, Berlin Prison Hospital, Friedrich-Olbricht-Damm 16, 13627 Berlin, Germany; 2 Institute of Forensic Psychiatry, Charité – Universitätsmedizin Berlin, Corporate Member of Freie Universität Berlin and Humboldt Universität zu Berlin, Oranienburger Str. 285, 13437 Berlin, Germany; 3 Department of Neurology with Experimental Neurology, Charité – Universitätsmedizin Berlin, Corporate Member of Freie Universität Berlin and Humboldt Universität zu Berlin, Hindenburgdamm 30, 12203 Berlin, Germany; 4 Department of Psychiatry and Neurosciences, Charité – Universitätsmedizin Berlin, Corporate Member of Freie Universität Berlin and Humboldt Universität zu Berlin, Hindenburgdamm 30, 12203 Berlin, Germany; 5 Berlin Institute of Health at Charité – Universitätsmedizin Berlin, BIH Biomedical Innovation Academy, BIH Charité Clinician Scientist Program, Charitéplatz 1, 10117 Berlin, Germany

**Keywords:** Catatonia, Chromosomal 18 deletion syndrome, Neurodevelopmental disorder, SARS-CoV-2 infection, Case report

## Abstract

**Background:**

Catatonia is increasingly diagnosed in neurodevelopmental disorders, yet most data focus on individuals with autism spectrum disorder. To our knowledge, catatonia has not been reported in association with chromosome 18 deletion syndrome.

**Case presentation:**

We describe a 19-year-old woman with a mosaic terminal 18q deletion (18q23) who developed severe catatonia following SARS-CoV-2 infection. She presented with mutism, stupor, negativism, posturing, rigidity, waxy flexibility, and withdrawal. Extensive neurological, infectious, and autoimmune workup, including cerebrospinal fluid analyses, revealed no abnormalities. Electroencephalography (EEG) showed intermittent frontotemporal right-dominant dysfunction, characterized by short clusters of high-amplitude polymorphic theta paroxysms without epileptiform discharges. Symptomatic treatment with lorazepam led to rapid initiation of speech within 24 h and full remission of catatonic features after four days. Follow-up EEG recordings at three and ten months were normal, and no recurrence occurred.

**Conclusions:**

This first reported case raises the possibility that individuals with the rare chromosome 18q deletion, including mosaic forms, may be vulnerable to catatonia particularly in the presence of systemic or environmental stressors such as viral infection or environmental change. Awareness of this potential association and early benzodiazepine treatment are critical to prevent complications and facilitate recovery.

## Background

Catatonia is a complex syndrome characterized by abnormal psychomotor activity, which can manifest as either increased or decreased motor activity or unusual behaviors. Historically, catatonia was considered a subtype of schizophrenia [1]. The eleventh revision of the International Classification of Diseases (ICD-11) now recognizes catatonia as a distinct clinical entity and differentiates between catatonia due to psychiatric illness, somatic conditions, or substance use [2]. According to the Diagnostic and Statistical Manual of Mental Disorders, Fifth Edition, catatonia may occur as a specifier of mood or psychotic disorders, associated with another medical, or of unspecified condition [3]. Early recognition and treatment are crucial, as effective interventions — such as benzodiazepines and electroconvulsive therapy — significantly improve outcomes. In contrast, delayed diagnosis or treatment is associated with increased morbidity and mortality [4]. Despite its clinical significance, catatonia remains underdiagnosed, particularly in the context of somatic illness [5]. A meta-analysis conducted in 2017 reported a prevalence of 9.2% across patients with psychiatric or medical conditions [6]. From a neurobiological perspective, catatonia is considered a disorder of cerebral motor network function [7]. Clinical electroencephalogram (EEG) abnormalities associated with catatonia and comorbid medical conditions were first described in the 1990s [8], and more recent studies indicate that EEG may assist in differentiating catatonia of general medical origin [9].

Although the precise neurobiological mechanisms underlying catatonia remain incompletely understood, the syndrome has been associated with a variety of medical conditions, including infectious diseases affecting the central nervous system. Emerging evidence suggests that immune dysregulation may play a role, although the mechanisms are unclear [10]. In contrast to case reports suggesting a possible link between COVID-19 and catatonic presentations [11], a systematic analysis by Luccarelli et al. did not observe an increased co-occurrence of COVID-19 and catatonia among hospitalized patients in the US [12].

Recent systematic reviews report that catatonia, especially in pediatric and neurodevelopmental populations, is associated with various genetic abnormalities. These include chromosomal copy number variants and other neurodevelopmental disorders affecting brain development. Converging findings suggest shared genetic vulnerability pathways [13–15]. In autism spectrum disorder, psychomotor abnormalities can overlap with catatonic symptoms, with an estimated prevalence of 10.4% [1] and may present with atypical features including regression [16]. Catatonia has been reported in patients with Down syndrome [17], and intellectual disability is recognized as a risk factor for delayed diagnosis of catatonia [18]. Rare cases describe stress-induced catatonic symptoms in children and adolescents, resembling dissociation-like states [19].

Despite the many known neurodevelopmental causes of catatonia and case reports linking it to rare genetic syndromes (e.g. 22q13 and 22q11 deletion syndromes [14]), an association with 18q deletion syndrome has, to our knowledge, not yet been reported. First described by de Grouchy in 1963, chromosomal 18 deletion syndrome arises from partial or complete deletions of chromosome 18 [20]. Clinical presentations are highly variable, but frequently include intellectual disability, speech difficulties, and dysmorphic features [21].

Here we report the first documented case, to our knowledge, of severe catatonia in a patient with mosaic terminal chromosome 18q deletion syndrome. The episode occurred following SARS-CoV-2 infection, suggesting that systemic or environmental stressors may precipitate catatonia in individuals with underlying neurodevelopmental vulnerability. This case expands the spectrum of genetic conditions associated with catatonia and highlights the importance of early recognition and prompt benzodiazepine treatment in medically ill patients with intellectual disability.

## Case presentation

Miss S., a 19-year-old woman living with her mother, had no family history of psychiatric disorders. Early childhood developmental delays in motor and speech skills prompted the diagnosis of mosaic terminal 18q deletion (18q23). The mosaic form was associated with a comparatively mild neurodevelopmental phenotype, as with initial support she attended regular school and, at the time of hospitalization, was completing high school. Her mother reported a largely independent lifestyle, with the ability to attend classes and manage routine self-care during her mother’s working hours. Other than a conductive hearing loss in the right ear, no functional impairments were reported. While she had no prior psychiatric diagnoses, episodes of social withdrawal and reduced speech production in unfamiliar settings and childhood bullying had been reported. Most recently, during a three-month school-organized stay abroad, she exhibited reduced speech production and social withdrawal but remained functional.

The patient was admitted for neurological evaluation following subacute behavioral changes, deterioration in general condition, and nonspecific symptoms including headache, nausea, and dizziness, progressively worsening over two weeks. On admission, she exhibited markedly reduced communication, limited to hand signals and vocalizations, and her mother reported decreased food and fluid intake. Twelve days prior, she had tested positive for SARS-CoV-2, necessitating isolation at home and disruption of daily activities.

Neurological examination revealed an awake but minimally cooperative patient with no spontaneous speech and no focal neurological deficits. An extensive diagnostic work-up - including cranial MRI, routine blood chemistry, inflammatory markers (including procalcitonin), microbiological testing (blood cultures), and comprehensive cerebrospinal fluid (CSF) analysis - revealed no abnormalities. CSF investigations included cell count and differentiation, protein and glucose levels, oligoclonal bands, and antibody indices (Reiber diagrams), as well as molecular diagnostics for infectious etiologies (including HSV, VZV, measles, mumps, and Borrelia burgdorferi). Importantly, a broad panel for autoimmune and paraneoplastic encephalitis was performed in CSF, including antibodies against NMDA receptor (NMDAR), AMPA1/2 receptors, GABA-B receptor, LGI1, CASPR2, DPPX, mGluR5, glycine receptor, dopamine-2 receptor, as well as classical onconeural antibodies (including Hu, Yo, Ri, Ma2/Ta, CV2/CRMP5, amphiphysin, and Tr/DNER). All tested antibodies, including NMDAR antibodies, were negative. In addition, thyroid autoantibodies (anti-TPO and anti-thyroglobulin) were assessed to exclude steroid-responsive encephalopathy associated with autoimmune thyroiditis (SREAT), with no pathological findings.

During the inpatient treatment, antiepileptic medication with Levetiracetam (2 × 500 mg) and Lamotrigine (25 mg) was initiated based on EEG findings (intermittent at least moderate bifrontotemporal dysfunction with right-sided predominance, characterized by short clusters lasting 1–2 s of high-amplitude polymorphic theta paroxysms at 4–6 Hz, interpreted as a sign of increased cortical excitability), despite the absence of epileptiform discharges and the lack of any clinically observed epileptic seizures. On the ward, fluctuating vigilance and reduced psychomotor activity were observed, and nursing staff described intermittent cooperation.

Psychiatric consultation was requested four days after admission due to persistent mutism, refusal to eat or drink, and negativism. Clinical assessment identified multiple catatonic signs: staring, stupor, mutism, grimacing, catalepsy, rigidity, waxy flexibility, automatic obedience, and withdrawal, with fluctuating negativism noted by staff. Bush-Francis Catatonia Rating Scale (BFCRS) score was 20. The patient met six positive criteria for catatonia according to DSM-5-TR and eight according to ICD-11 (see Table 1).

**Table 1:** Catatonic symptoms

Catatonic symptoms		Symptom present		Score
**DSM-5-TR**	**ICD-11**		**BFCRS**	
	**Decreased psychomotor activity**			
	Staring	yes	Staring	2
	Ambitendency	no	Ambitendency	0
Negativism	Negativism	yes	Negativism	2
Stupor	Stupor	yes	Stupor	2
Mutism	Mutism	yes	Mutism	3
			Withdrawal	3
	**Increased psychomotor activity**	no		0
Agitation		no	Psychomotor Agitation	0
			Impulsivity	0
			Combativeness	0
	**Abnormal psychomotor activity**			
Grimacing	Grimacing	yes	Grimacing	2
Mannerism	Mannerism	no	Mannerism	0
Posturing	Posturing	no		0
Stereotypy	Stereotypy	no	Stereotypy	0
	Rigidity	yes	Rigidity	1
Echolalia	Echophenomena	no	Echophenomena	0
Echopraxia		no		
	Verbigeration	no	Verbigeration	0
Waxy Flexibility	Waxy Flexibility	yes	Waxy Flexibility	3
Catalepsy	Catalepsy	yes	Posturing/ Catalepsy	1
			Automatic Obedience	1
			Mitgehen	0
			Gegenhalten	0
			Perseveration	0
			Autonomic Dysregulation	0
			Grasp Reflex	0

DSM-5-TR: Diagnostic and Statistical Manual of Mental Disorders, Fifth Edition, Text Revision

ICD-11: International Classification of Diseases, eleventh revision

BFCS: Bush-Francis Catatonia Rating Scale

Following psychiatric recommendation, benzodiazepine therapy (Lorazepam up to 4 mg/day, orally) was initiated, resulting in rapid and complete remission of catatonia. Due to negativism and mutism the initial Lorazepam doses of 1 mg were administered orally by the patient’s mother (legal guardian of the patient). Within one day, the patient resumed speech in short, complete sentences, began eating and drinking, and could be mobilized out of bed. Given the favorable tolerability, Lorazepam was increased to 4 × 1 mg daily. After four days, catatonic signs had fully resolved (BFCRS = 0), and the patient no longer fulfilled DSM-5-TR and ICD-11 criteria for catatonia. Lorazepam was tapered and discontinued over the following three days, with a total treatment duration of seven days.

Upon follow-up psychiatric consultation the patient retrospectively reported pronounced anxiety during the catatonic episode, particularly related to her inability to express herself verbally. In addition, unfamiliar hospital surroundings contributed to marked inner restlessness. She expressed relief at having regained the ability to speak and eat and stated her wish to be discharged home.

Outpatient follow-up demonstrated sustained remission. EEGs at three and ten months showed no abnormalities (see Table 2), and antiepileptic medication was tapered off between the months three and ten without recurrence of catatonic features.

**Table 2:** Electroencephalogram findings

Date*	State of consciousness	Rhythm	f	Amplitude	Abnormalities
Admission day	awake, fully alert and cooperative	alpha	11/s	up to 80µV	motion artifacts, no slowing of brain activity, signs of moderate fluctuation of vigilance, intermittent bifrontotemporal moderate dysfunction with signs of increased cortical excitability (high-amplitude polymorphic theta paroxysms)
7 days post‑admission	awake, fully alert and cooperative	alpha	9–10/s	up to 90µV	no slowing of brain activity, signs of mild fluctuation of vigilance, intermittent bifrontotemporal moderate to short sections of dysfunction with signs of increased cortical excitability
3‑month follow‑up	awake, fully alert and cooperative	alpha	10/s	up to 120µV	Signs of vigilance fluctuations with occasional reaching of sleep stage 2, intermittent moderate dysfunction in the bifrontocentral region with isolated sharp theta paroxysms, no definite epileptiform potentials.
10‑month follow‑up	awake, fully alert and cooperative	alpha	8–9/s	up to 90µV	No general abnormalities, intermittent moderate dysfunction in the right frontotemporal region, no epileptiform activity.

*To protect patient confidentiality, all dates are reported as relative time points; f: frequency

Subsequent follow-up contacts up to two years has not revealed any relapse. Miss S. completed high school and recently expressed the intention to pursue a vocational training.

## Discussion and conclusions

To our knowledge, no prior reports have described catatonia in association with terminal chromosome 18q deletion syndrome, including mosaic forms. Intercurrent SARS-CoV-2 infection and the unfamiliar hospital environment likely acted as stressors, suggesting a contribution to symptom onset and exacerbation.

A major strength of this case report is the comprehensive multidisciplinary diagnostic workup, including repeated neurological examinations, cranial MRI, extensive CSF analysis, serial EEG recordings, and standardized catatonia assessment using the Bush–Francis Catatonia Rating Scale. The clear temporal association between benzodiazepine initiation and rapid symptom resolution further supports the diagnostic validity of catatonia. However, several limitations must be acknowledged. As a single case report, causal inferences regarding the relationship between chromosome 18q deletion, SARS-CoV-2 infection, and catatonia cannot be established. Additionally, premorbid psychomotor and social characteristics related to the underlying neurodevelopmental disorder may complicate retrospective symptom attribution. In addition, the mosaic nature of the chromosomal deletion limits inferences regarding gene dosage effects and precludes generalization to non-mosaic 18q deletion syndromes.

Assessment of catatonia in patients with neurodevelopmental disorders is challenging due to symptom overlap. Hauptmann et al. recommend personalized baseline assessment and highlight six “red-flag” symptoms that may be overlooked: worsening stereotypies, new-onset refusal to eat, new or worsening incontinence, functional skill regression, increased self-injury, and purposeless agitation [16]. In this case, new-onset refusal to eat and functional skill regression were observed. The presence of mosaicism may further obscure baseline functioning, making third-party history an aid in differentiating baseline from new symptoms.

Growing evidence indicates that catatonia represents a transdiagnostic motor syndrome rather than a disorder confined to primary psychiatric illness [7]. Reports increasingly describe catatonia in the context of neurodevelopmental disorders, genetic syndromes, and acute medical conditions [10, 13, 14]. Findings that suggest SARS-CoV-2 infection as a potential precipitating factor for catatonia, possibly through inflammatory, neuroimmune, or stress-related pathways, remain inconclusive [11, 12]. Genetic vulnerability has been proposed as a modifier of catatonia risk, although evidence is still limited and largely based on case reports for rare chromosomal syndromes [13, 14]. Our findings align with this emerging literature and extend it by identifying chromosome 18q deletion as a previously unreported potential vulnerability factor.

Nonepileptic movement disorders have been rarely reported in adults with 18q deletion syndrome [22]. In patients with chromosomal 18 deletion, EEG abnormalities, including epileptiform activity and diffuse background changes, are reported in a small clinical study [23]. In contrast, our patient initially demonstrated increased cortical excitability rather than slowing [8, 9], which normalized on follow-up EEGs. Consistent with literature on underdiagnosis of catatonia in somatic contexts, understanding EEG abnormalities could aid earlier recognition [8, 9]. In our case, transient EEG abnormalities reflecting altered cortical excitability may represent a state rather than a primary etiological driver, particularly given their complete normalization on follow-up recordings.

The absence of structural brain abnormalities, infectious or autoimmune encephalitis, epileptiform EEG activity, and lack of any clinically observed epileptic seizures argue against alternative neurological explanations for the observed syndrome. In contrast, the rapid and complete remission following lorazepam administration strongly supports a diagnosis of catatonia. From a mechanistic perspective, we propose a stress–vulnerability model in which mosaic terminal chromosome 18q deletion may confer neurodevelopmental susceptibility, while acute systemic infection, social isolation, and environmental disruption act as precipitating factors. The mosaic nature of the patient’s 18q deletion may explain her relatively preserved premorbid functioning while still conferring subtle neurodevelopmental vulnerability that, under acute stress, contributes to dysregulation of motor and affective circuits.

This case highlights catatonia as a potentially reversible but underrecognized complication in individuals with rare neurodevelopmental syndromes. In patients with mosaic chromosome 18q deletion, acute medical illness and environmental stressors may precipitate severe catatonic states even in the absence of prior psychiatric disease. Diagnostic uncertainty may be compounded by overlapping baseline neurodevelopmental features and nonspecific EEG abnormalities. Prompt recognition of catatonia and early initiation of benzodiazepine treatment can result in rapid and complete recovery, preventing unnecessary diagnostic delay and prolonged morbidity. Clinicians should maintain a high index of suspicion for catatonia when patients with intellectual or developmental disabilities present with acute behavioral and psychomotor deterioration.

## Data Availability

The datasets analyzed during the current study are available from the corresponding author on reasonable request.

## Declarations

### Author information

Dr. Cosma is participant in the BIH Charité Clinician Scientist Program funded by Charité – Universitätsmedizin Berlin and the Berlin Institute of Health (BIH).

## Abbreviations

BFCRS: Bush–Francis Catatonia Rating Scale
CSF: Cerebrospinal fluid
COVID‑19: Coronavirus disease 2019
DSM‑5: Diagnostic and Statistical Manual of Mental Disorders, Fifth Edition
EEG: Electroencephalography / Electroencephalogram
ICD‑11: International Classification of Diseases, 11th Revision
MRI: Magnetic resonance imaging
SARS‑CoV‑2: Severe Acute Respiratory Syndrome Coronavirus 2
